# Is chest tube drainage necessary after subxiphoid thoracoscopic thymectomy?

**DOI:** 10.1186/s13019-020-01102-5

**Published:** 2020-04-22

**Authors:** Jiaduo Li, Guoyan Qi, Xiaohe Zhang, Xuguang Zheng

**Affiliations:** 1 Center of Treatment of Myasthenia Gravis Hebei Province, People’s Hospital affiliated to Hebei Medical University, Fangbei road No. 9, Shijiazhuang, 050011 Hebei Province China; 2 Center of Treatment of Myasthenia Gravis Hebei Province, First Hospital of Shijiazhuang, Fangbei road No. 9, Shijiazhuang, 050011 Hebei Province China

**Keywords:** VATS, Subxiphoid, Thoracoscopic thymectomy, Chest tube drainage, Myasthenia gravis

## Abstract

**Background:**

Subxiphoid thoracoscopic thymectomy has been increasingly performed in recent years. This study aimed to assess the differences in outcomes between subxiphoid thoracoscopic thymectomy with and without chest tube drainage.

**Methods:**

Overall, 205 subxiphoid thoracoscopic thymectomy operations were performed for myasthenia gravis, including 90 cases without and 115 cases with chest tube drainage. The clinical characteristics and perioperative outcomes of the patients were compared.

**Results:**

The patients included 112 women and 93 men, with a mean age of 41 years. Two patients in the group without and 5 patient in the group with chest tube drainage developed dyspnea. In the group without chest tube, 6 patients had residual pneumothorax or pleural effusion and had a thoracentesis after surgery (6/90). In the group with chest tube, 7 patients developed delayed pleural effusion and had a thoracentesis after chest tube removal (7/115). The patients in the group without chest tube drainage group yielded lower pain scores.

**Conclusions:**

The omission of chest tube drainage may be a feasible and safe choice for patients with myasthenia gravis undergoing subxiphoid thoracoscopic thymectomy, but further prospective studies are required.

## Introduction

Surgery is one of the most effective treatments for patients with myasthenia gravis (MG) [1]. Subxiphoid thoracoscopic thymectomy is less invasive than the lateral approach, so it has been increasingly used in thoracic surgery for cases of MG or anterior mediastinal tumors [2–5]. Chest tubes have been habitually placed in patients after thoracic surgery to evacuate fluids and air. Postoperative chest tube placement may increase the risk of infectious complications such as pneumonia or empyema, worsen the ventilation capacity, aggravate postoperative pain, and prolong hospitalization [6–8]. A few studies investigated cases of omission of chest drainage after lung resection several years earlier [9, 10]. Articles on the omission of chest tubes after thymectomy in MG are rare. In the present retrospective study, 210 subxiphoid thoracoscopic thymectomies for MG were reviewed to analyze the differences between patients with or without chest tubes, and evaluate the feasibility and safety of omitting chest tube drainage.

## Patients and methods

A retrospective review was conducted on the clinical data of all the patients who underwent subxiphoid thoracoscopic thymectomy for MG at the First Hospital of Shijiazhuang, affiliated with Hebei Medical University, between November 2017 and September 2019. This study was approved by the institutional review board of the First Hospital of Shijiazhuang. We initially performed subxiphoid thoracoscopic thymectomy in 2017 and, since then, routinely placed chest tubes after the procedure. Thereafter, we performed subxiphoid thoracoscopic thymectomy and omitted chest tube drainage in some cases in 2018. By the beginning of 2019, most cases of thymectomy for MG did not have chest drainage. The diagnosis of MG in all the patients was confirmed and clinically classified according to the standards of the Myasthenia Gravis Foundation of America (MGFA) [11]. All patients with MG had detectable antibodies against the acetylcholine receptor (AChR). We excluded patients with thymoma involving the innominate vein, pericardium, or lung. All surgeries were performed under thoracoscopy without conversion.

### Differences in surgical technique

The patients were placed in the supine position with their legs spread apart. Intubation with a single-lumen tube and mechanical ventilation were provided with the patient under general anesthesia. A skin incision (2–4 cm) was made 1 cm inferior to the xiphoid as an observation port. The surgeon’s finger was used to separate the retrosternal space to define the working space. Two 5-mm ports for the operation were created under the costal arch at the bilateral midclavicular lines with the guidance of the surgeon’s finger. A 30°, 10-mm thoracoscope was placed through the observation port. CO_2_ insufflation of the mediastinum was initiated at 6–8 cm H_2_O to amplify the anterior mediastinal space. An ultrasonic scalpel and thoracoscopic grasping forceps were inserted in the bilateral operating ports. The mediastinal pleura was dissected using an ultrasonic scalpel to reveal the bilateral phrenic nerves. The fat tissue at the bilateral cardiophrenic angle was then dissected. The bilateral mediastinal pleura was opened along the phrenic nerve, and the posterior border of the thymus was meticulously separated from the front edge of the superior vena cava and bilateral innominate veins to the thoracic outlet. The lobes of the thymus and mediastinal fat tissues were completely dissected from the pericardium, ascending aorta, and aorta–pulmonary window by using an ultrasonic scalpel. The thymic veins should be ligated and transected. After resection, the thymus along with the fat tissue was placed into a specimen bag and pulled out through the observation port.

In the chest tube drainage group, a small (*d* = 5 mm) chest tube was placed in the right side of the thoracic cavity through a 5-mm port after closing the other two incisions. The chest tube was then connected to the negative pressure drainage ball, and the lung was re-expanded by the anesthesiologist until no air leaked from the chest tube to the negative pressure drainage ball. In the group without chest tube drainage, a small (*d* = 5 mm) chest tube was placed in the right side of the thoracic cavity through a 5-mm port after closing the other two incisions. And then the chest tube was promptly removed and the drain site was sutured after the lung was re-expanded.

### Postoperative management and evaluation

After surgery, all patients received extubation after sobering from anesthesia and transferred to the care ward. Bedside chest radiography was routinely used to monitor postoperative bleeding, pneumothorax, and pleural effusion on the day of surgery and the following morning. Blood examination was performed on the first and third postoperative days (PODs). Postoperative analgesia with intravenous tramadol (400 mg qd) was given. Oral nonsteroidal analgesics and acetaminophen were also given once patients resumed oral intake 4 to 6 h after the operation. Postoperative pain was evaluated by a visual analog scale (VAS: 0 is completely pain free and 10 is hard to tolerate) after the procedure and on first three postoperative day [12]. The chest tube was removed when drainage was less than 100 ml/day with no air leakage.

### Statistical analysis

Continuous variables are presented as mean ± standard deviation (SD). Categorical variables are presented as number. The comparison between the groups for the continuous non-normal data was performed using the Mann-Whitney U test or Student’s *t* test according to normality. All statistical analyses were performed using SPSS ver. 21.0 (IBM SPSS Statistics, Chicago, USA).

## Results

### Clinical data of all the cases

The clinical and pathological data of all 205 cases are listed in Table 1. Of the patients, 93 were men and 112 were women, with a median age of 41 years (range, 9–77 years). The preoperative MGFA classification of cases were as follows: classes 1 (*n* = 13), 2a (*n* = 37), 2b (*n* = 100), 3 (*n* = 28), and 4 (*n* = 27). The pathological diagnoses included thymoma (*n* = 33), thymic hyperplasia (*n* = 110), thymic cyst (*n* = 5), and thymic involution or atrophy (*n* = 57; Tables 1, 2, 3).

**Table 1:** Characteristics of 205 cases

Factors	Total (*n* = 205)	Group A^a^ (*n* = 115)	Group B^a^ (*n* = 90)	p
Age				
Median and range (years)	41 (9–77)	41 (10–77)	40 (9–72)	0.545
< 50	158	92	66	
≥ 50	57	23	34	
Gender				
Women	112	61	51	0.607
Men	93	54	39	
MGFA Clinical Classification				
Class 1	13	7	6	0.722
Class2a	37	25	12	
Class2b	100	53	47	
Class3	28	13	15	
Class4	27	17	10	
Histopathology				
Thymoma	33	22	11	0.591
Thymic hyperplasia	110	58	52	
Thymic cyst	5	2	3	
Thymic involution or atrophy	57	33	24	

^a^Group A consists of patients with chest tube drainage; group B consists of patients without chest tube drainage

**Table 2:** Blood examination of 205 cases

Factors	Total (*n* = 205)	Group A^a^ (*n* = 115)	Group B^a^ (*n* = 90)	p
CRP^b^ (mg/L)				
PRD	2.9 ± 7.5	3.5 ± 9.3	2.2 ± 4.3	0.688
1POD	34.6 ± 22.8	38.5 ± 26.2	29.6 ± 16.2	0.020
3POD	15.4 ± 30.5	19.8 ± 39.0	9.8 ± 11.2	0.085
WBC^b^ (/μL)				
PRD	6500 ± 2400	6400 ± 2300	6700 ± 2500	0.427
1POD	11,600 ± 3700	11,700 ± 3900	11,600 ± 3400	0.947
3POD	7900 ± 2700	8000 ± 2800	7800 ± 2500	0.510
CK^b^ (U/L)				
PRD	76.6 ± 55.6	80.0 ± 61.7	72.3 ± 46.6	0.322
1POD	260.5 ± 146.4	255.6 ± 150.4	266.8 ± 141.7	0.591
3POD	60.5 ± 49.8	57.0 ± 51.8	65.1 ± 47.0	0.247
Hgb^b^ (g/L)				
PRD	133 ± 17	134 ± 17	132 ± 18	0.353
1POD	129 ± 16	132 ± 15	125 ± 17	0.005
3POD	129 ± 17	130 ± 16	128 ± 17	0.521

*CRP* C-reactive protein, *CK* creatine kinase, *Hgb* hemoglobin, *POD* postoperative day, *PRD* preoperative day, *WBC* white blood cell count

^a^Group A consists of patients with chest tube drainage; group B consists of patients without chest tube drainage

^b^Data expressed as mean ± standard deviation

**Table 3:** Perioperative results of 205 cases

Factors	Total (*n* = 205)	Group A^a^ (*n* = 115)	Group B^a^ (*n* = 90)	*p*
Operation time (min)^b^	145 ± 48	163 ± 51	122 ± 32	*P* < 0.0001
Blood loss (ml)^b^	40 ± 24	45 ± 26	34 ± 19	0.001
Duration of postoperative hospital stay (day)^b^	8.1 ± 1.6	8.3 ± 1.8	7.8 ± 1.2	0.095
Postoperative thoracic complications (effusion or pneumothorax)				
No thoracic complications	155	81	74	0.073
Not requiring thoracocentesis	37	27	10	
Requiring thoracocentesis	13	7	6	
Other complications				
Dyspnea	7	5	2	0.181
Phrenic nerve paralysis	1	1	0	
Death	0	0	0	
Duration of chest drainage (day)^b^		1.7 ± 0.6		
Chest tube drainage (ml)^b^				
POD1		139 ± 52		
POD2		102 ± 47		
Postoperative pain VAS score ^b^				
Surgery day		3.9 ± 0.8	3.1 ± 0.8	*P* < 0.0001
POD 1	3.5 ± 0.9	3.0 ± 0.8	2.6 ± 0.5	*P* < 0.0001
POD 2	2.8 ± 0.7	2.0 ± 0.8	1.4 ± 0.5	*P* < 0.0001
POD 3	1.5 ± 0.5	1.4 ± 0.5	1.6 ± 0.5	0.066

^a^Group A consists of patients with chest tube drainage; group B consists of patients without chest tube drainage

^b^Data expressed as mean ± standard deviation

The blood examination findings of all 205 cases are listed in Table 2. Regarding the postoperative laboratory data, creatine kinase and hemoglobin levels, and white blood cell count on the preoperative day (PRD), POD1 and POD3 were compared between the patients who underwent examination, and no significant differences were observed. Hemoglobin values were lower on POD1 in the group without chest tube drainage (*p* < 0.05). The C-reactive protein levels were significantly lower on POD1 in the group without chest tube drainage (*p* < 0.05).

The perioperative results of all the 205 cases are listed in Table 3. The operation time was shorter and the amount of blood loss was smaller in the group without chest tube drainage. The postoperative stay and pathological diagnoses were similar between the groups. Five patients in the group with and two patient in the group without chest tube drainage developed dyspnea and recovered after receiving mechanical ventilation. In the group with chest tube, 7 patients developed delayed pleural effusion and had a thoracentesis after chest tube removal (7/115). In the group without chest tube, six patients had residual pneumothorax or pleural effusion and had a thoracentesis after surgery (6/90). The duration of chest tube drainage was 1.7 ± 0.6 days in the group with chest tube drainage. The mean chest tube drainage was 139 ml on POD 1 and 102 ml on POD 2. In addition to the POD3, comparing the group with chest tube drainage, patients in the group without chest tube drainage had less pain (VAS for Surgery day, 3.1 ± 0.8 vs.3.9 ± 0.8, *P* < 0.05; VAS for POD1, 2.6 ± 0.5 vs. 3.0 ± 0.8, *P* < 0.05; VAS for POD2, 1.4 ± 0.5 vs.2.0 ± 0.8, *P* < 0.05). No in-hospital deaths occurred.

## Discussion

The thymus plays a key role in AChR-mediated MG [13], and thymectomy is a treatment option for patients with this subtype. The lateral approach has been considered as a standard procedure for video-assisted thoracoscopic surgery thymectomy [14–16]. The subxiphoid approach leads to a less invasive thoracoscopic thymectomy than the lateral approach [5]. The use of chest tubes after thymectomy as a routine and universal practice is crucial to monitor bleeding, air leakage, and pleural effusion. Most surgeons believe that leaving the chest tube after surgery will enable monitoring of early postoperative complications such as pneumothorax or retained hemothorax. Xu et al. reported their experience in omitting chest tube drainage after subxiphoid thoracoscopic thymectomy [17]. However, no case-control studies have been conducted on patients with MG who underwent subxiphoid thoracoscopic thymectomy without chest drainage.

The main pitfall of omitting chest tube placement was dyspnea. Five patients in the group with and two patients in the group without chest tube drainage developed dyspnea and recovered after receiving mechanical ventilation. The most common complications were effusion or residual pneumothorax. In the group without chest tube, six patients had residual pneumothorax or pleural effusion and had a thoracentesis after surgery (6/90). To obtain a satisfactory surgical view, an artificial pneumothorax was established at 6–8 cm H_2_O to amplify the anterior mediastinal space during the operation, and the lung was re-expanded by the anesthesiologist until no air leakage was confirmed before closing the incision [18]. In thymectomy, the lung is intact in most cases, and the main cause of pneumothorax after the operation is insufficient exhaust before suture incision. In the group with chest tube, 7 patients developed delayed pleural effusion and had a thoracentesis after chest tube removal (7/115). So the chest tube drainage did not prevent late effusions. There was no statistically significant difference in postoperative complications between the two groups. This suggests that omitting chest tube placement does not increase the risk of postoperative complications.

We used the three-portal “subxiphoid and subcostal arch” approach reported by Lu et al. [4]. The thoracoscopic subxiphoid approach may prevent damage to the intercostal nerves, reducing postoperative pain [5]. However, patients who underwent subxiphoid thymectomy still suffered postoperative pain. The placement of a chest tube is considered to enhance postoperative pain [7, 19]. When the subjective evaluation of pain scores was evaluated, pain sensations were less in the group without chest tube drainage, especially on surgery day and the first two postoperative days. The strategy of omitting the chest tube could minimize postoperative pain and improve recovery.

Owing to the significant improvements in the instruments used in thoracoscopic surgery, only little bleeding occurs in thymectomy. In this study, the chest drainage group had a slightly longer operation time and slightly greater intraoperative blood loss than the non-chest drainage group, which may be due to learning curve of subxiphoid thoracoscopic thymectomy. We started subxiphoid thoracoscopic thymectomy with chest tube drainage in 2017, and then performed thymectomy omitting chest tube in 2018 and thereafter. The difference of operation time and blood loss may be the result of learning curve, since blood loss may increase with the increase of operation time. We believed that omission of chest tube placement is safe if the intraoperative hemostasis is reliable in thoracoscopic thymectomy. However, without a chest tube, postoperative bleeding cannot be directly observed. Bedside chest roentgenography and routine blood examinations were indispensable for these patients, and pneumothorax hemorrhagic effusion may be diagnosed without delay.

We acknowledge several limitations of the study, including its retrospective design and the choice to place a drainage or not was surgeon-related. The cases of indwelling chest tube were mostly in the early stage of the study, while the cases of omitting chest tube were mostly in the later stage of the study. This difference may bias clinical outcomes as the learning curve changes. In selecting cases, we excluded patients with thymoma involving the innominate vein, pericardium, or lung. The results should be strictly interpreted when applying this procedure to future patients.

## Conclusion

In summary, the strategy for the omission of chest tube drainage in patients undergoing subxiphoid thoracoscopic thymectomy is a safe method that does not increase the risk of adverse events. This procedure may be an alternative for certain patients with MG undergoing thoracoscopic thymectomy. Owing to the limitation of our retrospective study, further prospective studies are needed to evaluate clinical outcomes and safety.

## Data Availability

The datasets generated and analyzed during the current study are available from the corresponding author on reasonable request.

## Abbreviations

MG: Myasthenia gravis
MGFA: Myasthenia Gravis Foundation of America
AChR: Acetylcholine receptor
SD: Standard deviation
POD: Postoperative day
PRD: Preoperative day
